# Towards the Preparation of a Hydrogel from Lyophilisates of the *Aloe arborescens* Aqueous Extract

**DOI:** 10.3390/pharmaceutics14071489

**Published:** 2022-07-18

**Authors:** Kamil Pawłowicz, Magdalena Paczkowska-Walendowska, Tomasz Osmałek, Judyta Cielecka-Piontek

**Affiliations:** 1 Department of Pharmacognosy, Poznan University of Medical Sciences, Rokietnicka 3, 60-806 Poznan, Poland; kamil.pawlowicz@europlant-group.pl (K.P.); mpaczkowska@ump.edu.pl (M.P.-W.); 2 Phytopharm Klęka S.A., Klęka 1, 63-040 Nowe Miasto nad Warta, Poland; 3 Department of Pharmaceutical Technology, Faculty of Pharmacy, Poznan University of Medical Sciences, Grunwaldzka 6, 60-780 Poznan, Poland; tosmalek@ump.edu.pl

**Keywords:** *Aloe arborescence*, hydrogel, antioxidant activity, anti-hyaluronidase activity

## Abstract

*Aloe* gel is a medicinal raw material with proven pharmacological activity. The health-promoting properties of other species of *Aloe* upon topical application prompted us to develop a formulation for the topical application of *A. arborescence* species. As a result of the gel preparation from the aqueous lyophilized extracts of three-year-old leaves of *A. arborescence*, no changes in the composition of the content of aloins A and aloenin A were found. The potential to neutralize free radicals was tested using DPPH and CUPRAC techniques, which confirmed the anti-radical activity of the lyophilisate. Screening of the inhibition of enzymes, the hyperactivity of which is associated with adverse changes in the skin of a pro-inflammatory nature, was performed. Importantly, using the PAMPA SKIN model, the possibility of the penetration of selected extract compounds (aloin A and aloenin A) through the skin was proven. Then, two formulations were prepared based on sodium alginate and hydroxypropyl methylcellulose (HPMC) and the hydrogels were characterized (rheological analysis, drug release profiles, permeability, and stability studies). HPMC-based hydrogel was the one with a targeted release of active substances and greater stability. *Aloe arborescens* hydrogel matrices seem to be a promising treatment strategy for inflammatory surface damage based on “green technology” at the stage of extract preparation and development of the drug form for topical application.

## 1. Introduction

Plant materials contain active compounds that may show synergistic action. There are many reports in the literature studying the possibility of plants causing pharmacological effects due to active compounds with different chemical structures, for example, the activity of the plant material from *Cannabis* sp. or *Calendula* sp. [1,2]. The critical step in obtaining the appropriate composition of compounds is their proper extraction. Organic solvents are still often used in its implementation. When obtaining a specific extraction efficiency, one should remember the consequences of their use, such as the possibility of their residues influencing living cells or environmental contamination related to their use [3,4].

Ecologically friendly extraction techniques include those with the use of water as the extraction factor [5,6] or the application of procedures that are safe for the natural environment, e.g., supercritical fluid extraction (SFE) [7,8]. The number of works reporting ecological extraction methods is constantly increasing. Srivastava and Gupta described the biological activity of aqueous extracts of chamomile flowers towards anti-proliferative and apoptotic activity in various human cancer cells [9]. The number of cases using supercritical extraction as a green technique to obtain extracts with a composition ensuring biological activity is also increasing. Recently, an example of the use of SFE in getting extracts from medicinal cannabis has been reported [10].

One of the valued ingredients with a multi-pharmacological effect is the leaves of various species of *Aloe*. Aloe leaf gel is widely used in the pharmaceutical and cosmetic industries. The rationale is the composition of the active compounds. *Aloe* spp. contain almost 100 different compounds, including polysaccharides (e.g., acemannan, glucomannan), glycoproteins (e.g., aloctin), anthraquinones (e.g., aloin, emodin, aloenin), phenolic acids (e.g., salicylic acid), fatty acids (e.g., lupeol and campesterol), enzymes (e.g., amylase, catalase, and peroxidase), and others such vitamins and minerals [11,12]. It has been proven that the composition of these compounds can have anti-inflammatory and antiseptic properties and is therefore helpful in the healing of wounds and burns [12,13,14]. It is worth considering the exact mechanism by which aloe gel improves wound healing. Its therapeutic properties are linked to glucomannan, a molecule that affects fibroblast growth factor and boosts the activity and proliferation of these cells, resulting in increased collagen formation and secretion [15]. Aloe gel has also been found to not only increase the amount of collagen in wounds but also to affect collagen composition and cross-linking, promoting wound healing [16]. It also had significant stimulatory effects on cell proliferation and migration of both fibroblasts and keratinocytes [17]. According to clinical trials, aloe gel has also been used to prevent skin ulcers and cure burn wounds, surgical wounds, cracked nipples, genital herpes, psoriasis, and chronic wounds such as pressure ulcers [16,18].

When considering the possibility of topical and skin application of aloe extracts, it is worth paying attention to the pharmaceutical form of providing extracts in the form of hydrogels. Hydrogels appear to be one of the most promising classes of biomaterials due to their good physicochemical features, biocompatibility, and planned interaction with biological surroundings. They can be successfully used in the technology of highly controlled active substance release systems to the target site for a long period of time [19].

While the activity of other aloe species is well understood, there is still little information about the composition of the active compounds in *A. arborescens* and its biological activity. Therefore, our previous research has focused mainly on evaluating the performance of the *A. arborescens* garland. Leaves from control crops were used as the raw material for the tests. In the first phase of the research, we compared the composition of leaves obtained from one, two, three and four-year-old plants. Leaves obtained from three-year-old plants were indicated as the most worthwhile as they had the highest total phenolic concentration and total antioxidant activity [20]. For them, ethanol extracts were obtained, for which the safety of their use and effectiveness in relation to fibroblasts was also proven [21]. Bearing in mind the eco-friendly development of the Aloe leaf hydrogel, in this study, we have shown that aqueous extracts of *A. arborescens* allow for the obtention of biological activity, inhibiting enzymes whose overexpression promotes skin lesions. Additionally, the hydrogel has been proposed as a convenient and modern form of topical administration with a prolonged release of active substances and effective local action.

## 2. Materials and Methods

### 2.1. Plant Material

The leaves of three-year-old *Aloe arborescens* plants were obtained from plants from the controlled cultivation of Phytopharm Klęka S.A. (Nowe Miasto nad Wartą, Poland).

### 2.2. Chemicals and Reagents

Aloin A and Aloein A (phyproof^®^ Reference Substance) were obtained from Sigma-Aldrich (Poznan, Poland), as well as all reagents for activity assays: (Sigma-Aldrich). 2,2-Diphenyl-1-picrylhydrazyl (DPPH), neocuproine, acetate buffer, copper(II) chloride (CuCl_2_·H_2_O), kaempferol, sodium chloride, bovine serum albumin, hexadecyltrimethylammonium bromide (CTAB), hyaluronic acid (HA), hyaluronidase, sodium hydroxide, tyrosinase, L-DOPA, hydroquinone, Tris-HCl buffer, galantamine, acetylocholinesterase (AChE) and butyrylcholinesterase (BChE), 5,5-dithio-bis-(2-nitrobenzoic acid (DTNB), and phosphate buffer. The hydration solution and acceptor Prisma™ buffer were obtained from Pion Inc. (Billerica, MA, USA), whereas HPLC grade methanol and water were obtained from Merck. High-quality pure water and ultra-high quality pure water were prepared using a Direct-Q 3 UV Merck Millipore purification system.

### 2.3. Preparation and Analysis of Aloe Gel Lyophilizate and Extract–Preformulation Studies

#### 2.3.1. Aloe Aqueous Gel Lyophilization and Extract Preparation

To obtain aloe gel in a stable form, the leaves were thoroughly washed and dried, then the base of the leaves was cut off and separated the inner part from the peel. Then, the aloe gel was frozen at −20 °C and subjected to the lyophilization process (Heto PowerDry PL3000 Freeze Dryer, Thermo Scientific, Waltham, MA, USA) for 72 h under vacuum conditions at a temperature of about −55 °C.

This obtained lyophilized aloe gel was rehydrated with water in the ratio 5:95 (*w*/*v*) at the temperature 40 °C in an ultrasonic bath to a gel close to its fresh state to obtain water-soluble compounds.

#### 2.3.2. Determination of Active Compound Content

The concentrations of main active compounds (aloin A and aloenin A) were determined by using the HPLC-Diode-Array Detection method (equipment LC system, Dionex Thermoline Fisher Scientific) with Chromeleon software version 7.0) described previously [20]. Briefly, separations were performed on a LiChrospher RP-18 column, 5 μm particle size, 250 mm × 4 mm LiChroCART^®^ 250-4 (Merck, Darmstadt, Germany). The detection was performed using a diode array detector at a wavelength maximum (*λ*_max_) of 295 nm. The following mobile phase was composed of water (A) and methanol (B) with a gradient elution: 0–35 min, 35–95% B; 35–40 min, 95% B; 40–45 min, 35 B with a flow rate of the mobile phase set at 1.0 mL/min and the column temperature was maintained at 30 °C.

The presence of aloin A and aloenin A in the lyophilizate and extract was confirmed by comparison of the retention time and UV spectra of analyzed substances with their reference standards.

#### 2.3.3. Antioxidant Activity

##### Assay with 2,2-Diphenyl-1-picrylhydrazyl (DPPH)

The DPPH assay was conducted according to Paczkowska-Walendowska et al. [22]. 25 μL of water extracts at concentrations 4.17–66.67 mg plant material per mL were mixed with a 175 μL of 0.2 mmol/L DPPH solution. The reaction mixture was shaken and incubated in the dark at room temperature for 30 min. Absorbance was measured at 517 nm against the blank (25 μL of water and 175 μL of methanol). The control sample contained 25 μL of water and 175 μL of DPPH solution. Six replicates were performed for each assay. The percent of DPPH scavenging activity was calculated according to the following formula:

$$DPPH_{\mathrm{scavenging}\;\mathrm{activity}\;}\left(\%\right)=\frac{A_{0}-A_{1}}{A_{0}}\times 100\%$$

where *A*_0_ is the absorbance of the control and *A*_1_ is the absorbance of the sample.

##### Cupric Reducing Antioxidant Capacity (CUPRAC) Assays

The CUPRAC assay was conducted according to Paczkowska-Walendowska et al. [22]. The solutions of the CUPRAC reagent included equal parts of 7.5 mM neocuproine solution in 96% ethanol, acetate buffer (pH = 7.0), and 10 mM copper (II) chloride solution. Briefly, 50 μL of water extract was mixed with 150 μL of CUPRAC solution, mixed, and incubated in the dark condition at room temperature for 30 min. Then, absorbance was measured at 450 nm against the control (50 μL of water and 150 μL of CUPRAC reagent). The analysis was performed in six replicates. The results were expressed as the IC_0.5_, which corresponds to the extract concentration required to obtain the absorbance value 0.5.

#### 2.3.4. Anti-Hyaluronidase Activity

The inhibition of hyaluronidase was determined by a turbidimetric method described by Studzińska-Sroka et al. [23]. Twenty-five µL of hyaluronidase enzyme (30 U/mL of acetate buffer pH 7.0), 25 µL of acetate buffer (50 mM, pH 7.0, with 77 mM NaCl and 1 mg/mL of bovine albumin), 15 µL of acetate buffer (pH 4.5), and 10 µL solutions of the gel were combined in order to produce reagent mixtures. All the reaction mixtures were incubated at 37 °C for 10 min. Next, 25 µL of hyaluronic acid solution (HA; 0.3 mg/mL of acetate buffer pH 4.5) was added and incubated at 37 °C for 45 min. The undigested HA was precipitated with the addition of 200 µL 2.5% CTAB in 2% NaOH. The mixture was kept at room temperature for 10 min. Turbidance of the reaction mixture was measured as the absorbance at 600 nm (Multiskan GO 1510, Thermo Fisher Scientific, Vantaa, Finland). Kaempferol was used as the positive control (final concentration 0.5–1.0 mg/mL).

Also, five blank samples have been prepared:

- blank 1: enzyme and hyaluronic acid solution were replaced with acetate buffer (25.0 µL) and the test solution was replaced with the water (10.0 µL),
- blank 2: the enzyme solution was replaced with acetate buffer (25.0 µL) and the test solution was replaced with the water (10.0 µL),
- blank 3: the test solution was replaced with water (10, 0 µL),
- blank 4: hyaluronic acid solution replaced with acetate buffer (25.0 µL),
- blank 5: the enzyme solution was replaced with acetate buffer (25.0 µL).

The percentage of inhibition of hyaluronidase activity was determined according to the following equation:

$$I\%=\frac{\left(A_{S}-A_{B4}\right)-\left(A_{B3}-A_{B1}\right)}{\left(A_{B5}-A_{B4}\right)-\left(A_{B3}-A_{B1}\right)}\times 100\%$$

where *I*% is the % inhibition of hyaluronidase, *A_S_* is the absorbance of the sample, *A_B_*_1_ is the absorbance of blank 1, *A_B_*_3_ is the absorbance of blank 3, *A_B_*_4_ is the absorbance of blank 4, and *A_B_*_5_ is the absorbance of blank 5.

#### 2.3.5. Anti-Tyrosinase Activity

Seventy-five μL of phosphate buffer (pH 6.8), 50 μL of 192 U/mL tyrosinase solution, and 25 μL of appropriate concentrations of the extract were combined in order to produce reagent mixtures. All the reaction mixtures were incubated at 37 °C for 10 min. Next, 25 µL of HA (0.3 mg/mL of acetate buffer pH 4.5) was added and incubated at room temperature for 8 min. Then, 50 μL of 2 mM/L L-DOPA solution was added to the mixture and incubated again at room temperature for 20 min. The absorbance was measured at 475 nm (Multiskan GO 1510, Thermo Fisher Scientific, Vantaa, Finland). Hydroquinone was used as the positive control (final concentration 5.0 mg/mL).

The tyrosinase inhibitory activity was calculated according to the formula below:

$$I\%=\left[\frac{A_{PK}-A_{PB}}{A_{PK}}\right]\times 100\%$$

where *I*% is the % inhibition of tyrosinase, *A_PK_* is the control sample absorbance−control background absorbance, *A_PB_* is the test sample absorbance − sample background absorbance.

#### 2.3.6. Effect on Acetylocholinesterase (AChE) and Butyrylcholinesterase (BChE) Activity

In order to perform the analysis, the following were measured into each of the wells of the plate: 60 μL of Tris-HCl buffer (0.05 M/L), then 5 μL of galantamine solution or test extract of the appropriate concentration and 30 μL of AChE or BChE enzyme solution (0.2 U/mL) were added to the test samples and incubated for 5 min at room temperature. Then, 30 µL of ATCI or BTCI solution (1.5 mM/L) and 125 µL of DTNB solution (0.3 mM/L) were added to each well and the plate was incubated for 20 min for AChE or 30 min for BChE at room temperature. The absorbance measurement at 405 nm. In the blank for the test samples, the enzyme solution was replaced with a buffer; in the control, the medium used for the extraction was used instead of the test solution; in the blank for the control, in addition to changing the extract to a pure medium, the enzyme was replaced with an appropriate volume of the buffer. The result was calculated according to the formula:

$$enzyme\;inhibition\;\left(\%\right)=100-\frac{(A_{S}-A_{SB})\times 100}{A_{C}-A_{CB}}$$

where *A_S_* is the absorbance of the sample, *A_SB_* is the absorbance of the sample’s blank, *A_C_* is the absorbance of the control, and *A_CB_* is the absorbance of the control’s blank.

#### 2.3.7. Permeability Assay

Permeability of aloe gel active compounds through artificial biological membranes was investigated by using the PAMPA™ (skin parallel artificial membrane permeability assay) SKIN assays (Pion Inc.) as received by the manufacturer, without further evaluation of the membrane permeability. In brief, the PAMPA™ model consists of a two-chamber PAMPA™ sandwich composed of two 96-well plates, with donor on the bottom plate and acceptor on top plate. In the case of the Skin model, before use, pre-coated Skin PAMPA™ sandwich plates were hydrated overnight by placing 200 μL of the hydration solution in each well (Hydration Solution, Pion Inc.). Standards (aloenin A and aloin A) were dissolved in donor solutions. The acceptor plates were loaded with an acceptor Prisma buffer with pH 7.4. The plates were put together and incubated under the following conditions: temperature of 32 °C for 5 h with continuous stirring at 60 rpm. Each experiment was repeated at least three times. The amount of permeated active compounds was determined using the HPLC method.

The apparent permeability coefficients (*P_app_*) were calculated from the following equation:

$$P_{app}=\frac{-ln\left(1-\frac{C_{A}}{C_{equilibrium}}\right)}{S\times \left(\frac{1}{V_{D}}+\frac{1}{V_{A}}\right)\times t}$$

where *V_D_* is the donor volume, *V_A_* is the acceptor volume, *C_equilibrium_* is the equilibrium concentration $C_{equilibrium}=\frac{C_{D}\times V_{D}+C_{A}\times V_{A}}{V_{D}+V_{A}}$, *C_D_* is the donor concentration, *C_A_* is the acceptor concentration, *S* is the membrane area, and *t* is the incubation time (in seconds).

### 2.4. Formulation Studies

For hydrogel formulations, the following composition was proposed (Table 1).

To obtain a hydrogel, sodium alginate or HPMC was added to the distilled water and stirred for 1 h with a magnetic stirrer, maintaining the temperature at 40 °C or 65 °C, respectively. After this time, the heat was turned off and hyaluronic acid was added and stirred for another hour. Finally, lyophilized aloe gel was added and mixed again for 1 h.

#### 2.4.1. Rheological Analysis

Examination of the rheological properties of formulation 1 and formulation 2 was performed at a temperature of 32.0 ± 0.5 °C with the use of HAAKE™ RheoStress1 (Thermo Scientific Corp., Waltham, MA, USA) rotational rheometer equipped with parallel plate geometry (PP35Ti; diameter: 35 mm, gap size: 1 mm).

In flow behavior studies, the investigated samples were subjected to shearing at controlled rate (CR) and controlled stress (CS) modes. In CR mode, the shear rate increased from 1.0 to 200.0 1/s in 100 s. In CS mode, the shear stress value increased from 1.0 to 200.0 Pa in 100 s.

In the oscillatory stress sweeping assay, the samples were subjected to the increasing oscillatory stress in the values from 1.0 to 50.0 Pa under a constant frequency of 1 Hz. Storage (G′) and loss (G″) moduli were plotted as a function of oscillatory stress.

#### 2.4.2. Drug Release Profiles

The in vitro release experiments were performed for the formulation 1 and 2 with the use of vertical Franz diffusion cells (SES GmbH–Analysesysteme, V6A-02 model), each containing 8 mL of acceptor solution (phosphate buffer; pH = 7.4). The cells were equipped with regenerated cellulose membranes (Visking^®^ dialysis tubing, SERVA Electrophoresis GmbH, Heidelberg, Germany) with pore diameter ca. 25 Å. Gel samples (1.0 mL) were placed at the donor compartment and spread evenly on the surface of the artificial membrane (immersed in the acceptor fluid at 32.0 ± 0.5 °C for 24 h before the experiment). Each gel sample (1.0 mL) contains 0.04 mg of aloin A and 0.03 mg of aloenin A. The solubility of aloin A is 1 mg/mL while aloenin A is freely soluble in the water; therefore, sink conditions were retained. The effective diffusion area of the employed cells was 0.999 cm^2^. The temperature of the receptor fluid was set at 32.0 ± 0.5 °C. The samples (1.0 mL) were taken from the acceptor compartment after every hour, up to 6 h, and replaced immediately with an equal volume of fresh acceptor fluid. The concentrations of active compounds in the collected samples were determined with the UHPLC-DAD method described above.

#### 2.4.3. Biological Assays

Antioxidant activity using DPPH assay was performed according to Section 2.3.3.

Anti-hyaluronidase and anti-tyrosinase activities of formulation 1 and 2 were also tested. An appropriate volume of formulations 1 and 2 was added to the reagent mixture and thoroughly dispersed according to Section 2.3.4 and Section 2.3.5. The percentage of inhibition was converted to the content of the lyophilized aqueous gel to make the results comparable.

A permeability test was performed according to Section 2.3.7. Formulation 1 and 2 were dispersed in donor solution. The other conditions were as described above.

#### 2.4.4. Stability Test

Both original lyophilized aloe aqueous gel (semi-product) as well as formulations 1 and 2 have undergone stability testing. The samples were placed in a stability chamber with intermediate conditions (temperature: 30 ± 2 °C, humidity: 65 ± 5% RH). The content of active ingredients was assessed at the time points 0, 10, and 20 days by using the HPLC method.

### 2.5. Statistical Analysis

Statistical analysis was carried out with Statistica 13.3 software (Cracow, Poland). The normality of the results was checked using the Shapiro–Wilk test. The differences among the mean values were tested using the ANOVA test with post hoc Tukey’s range test for multiple comparisons (when comparing three or more groups, e.g., in the case of biological activity of extracts) or using the Student’s *t*-test (when comparing two groups, e.g., in the case of permeability assay of formulations). Differences between groups were considered to be significant at *p* < 0.05.

## 3. Results and Discussion

**Figure 1:**
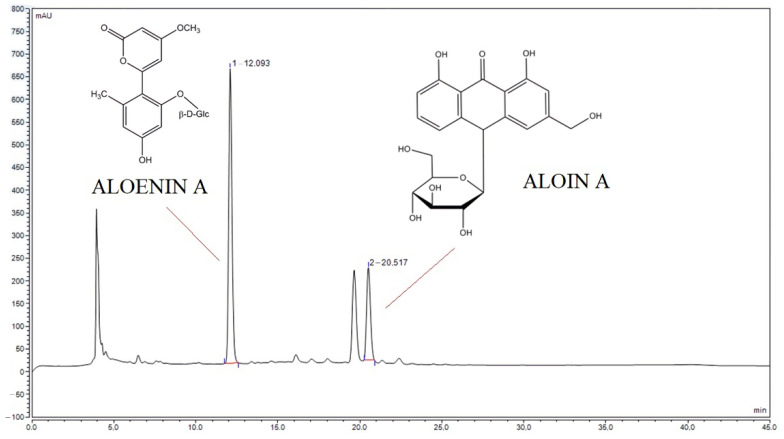
The HPLC chromatogram of lyophilized aloe gel with selected patterns of aloenin A and aloin A.

The literature reports on the biological properties of alcoholic aloe extracts [20]. Rather than helping wounds heal, alcohol can cause pain and irritation and slow down the healing process, so, unfortunately, the use of alcoholic extracts in the treatment of hard-to-heal wounds may be limited [24]. Therefore, the aim was to prepare water extracts and assess their biological activity profile. To prepare the extracts, three-year-old leaves were used because in our earlier study, it was shown that they have the highest content of active compounds with the most potent antioxidant properties [20]. In order to assess the qualitative and quantitative composition, the previously developed HPLC method was used, in which the content of aloin A and aloenin A was evaluated (Figure 1) [20]. Identification of the additional peaks was not possible due to limited access to the analytical standards. Aloin A and aloenin A contents were 1.49 mg and 1.19 mg per 1 g of lyophilizate, respectively.

The next stage of the research was to evaluate the properties of the prepared aqueous aloe extract. The DPPH and CUPRAC methods were used in the assessment of antioxidant properties (Table 2). It was shown that the prepared extract is even more active than the alcoholic extract, with IC_50_ = 22 vs. 37 mg/mL for water and alcoholic extract for CUPRAC assay, respectively [20]. The antioxidant activity does not only result from the presence of polyphenolic compounds; hence, a synergy between the action of polyphenols and anthranoid compounds can be suggested. This is confirmed by the previously obtained test results, where rind extracts, much richer in anthranoid compounds, show higher antioxidant activity [25].

When considering the use of aloe arborescence extract in the treatment of hard-to-heal wounds, an anti-inflammatory effect is also a critical property. For this purpose, the ability of the extract to inhibit the hyaluronidase enzyme was assessed. The presence of hyaluronic acid supports and accelerates the healing process of both superficial and deep wounds of various origins, resulting in faster wound healing, while hyaluronidase is an enzyme that catalyzes the degradation of hyaluronic acid [26]. Therefore, the hyaluronidase inhibitory properties of aloe gel water extract were assessed (Table 3). The hyaluronidase inhibition by *A. arborescens* extract is lower (7.03% at a concentration of 66.67 mg/mL, Table 3) than that of the other varieties: *A. camperi*–771.78 µg/mL, *A. percrassa* latex—664.47 µg/mL [27], and *A. arborescens* ethanol extract–660.00 µg/mL [21]. The effect of the prepared lyophilized gel was weaker than the positive control, kaempferol (IC_50_ = 713 µg/mL). Such a significant difference in activity may result from the method of obtaining the extract; in the above, the extraction mixture was ethanol or ethanol-water (7:3 *v/v*), while in this test, it was water. It is believed that the inhibition of skin-related enzymes (hyaluronidases, but also elastase, collagenase, and tyrosinase), which mainly act as spreading agents that reduce the tension in the connective tissue, is a crucial strategy for good skin integrity and wound healing [28]. While there are no direct literature reports on the effect of aloin on hyaluronidase, there is information about its anti-collagenase activity [29]. It can be supposed that aloin will also be responsible for the anti-hyaluronidase activity of the said extracts. It is aloin that is one of the compounds that determine the anti-inflammatory effect of the plant material, probably through the blocking of iNOS and COX-2 mRNA expression [30]. Furthermore, deregulation of hyaluronic acid metabolism is one of the features of diabetes mellitus, where the leading cause of this phenomenon is elevated glucose levels. This phenomenon is the cause of poor wound healing and many of its complications observed in diabetics [31]. Therefore, it can be concluded that aloe extract, which is characterized by high hyaluronidase-inhibiting properties, may become a promising treatment strategy for these wounds.

On the other hand, tyrosinase is an enzyme that plays a role in the formation of pigments such as melanin. Overactivity of the tyrosinase enzyme leads to hyperpigmentation and is associated with aging [32]. Therefore, its inhibitors can be attractive skin-lightening agents [33] and they can be used in wound and scar treatment. Therefore, the anti-tyrosinase activity of water aloe gel extracts was also assessed (Table 3). Interestingly, aloe gel extract at a concentration of 66.67 mg/mL inhibits tyrosinase by 14.43% (Table 3) and the extract described in the literature at a concentration of 600 μg/mL does not, so the effect is dose dependent. The effect is weaker than the positive control, hydroquinone (42% at a concentration 5 mg/mL). Whereas Gupta et al. showed that methanolic extracts at a concentration of 6 mg/mL inhibits tyrosinase by 41.18% [34]; thus, a significant influence of the extraction solvent on the activity is apparent. Another study conducted by Jones et al. showed that aloesin, another anthranoid compound isolated from the Aloe plant, modulates melanogenesis by competitive inhibition of tyrosinase at the site of dihydroxyphenylalanine oxidation, inhibiting melanin synthesis in vitro, and its action is dose dependent [35,36].

When considering the use of aloe gel extract in patients with diabetes, it is worth noting that acetylcholinesterase deficiency contributes to neuromuscular junction dysfunction in diabetic neuropathy [37]. Therefore the influence of the prepared extract on AChE and BChE activity was also examined (Table 4). Some activity was shown against both enzymes; however, it is several hundred times weaker than the standard, whereas in the case of peripheral neuropathies, preservation of enzyme activity is indicated. The inhibitory activity of ethanol extracts is much higher [38], which proves the suitability of using water extracts as indicated in this study.

**Figure 2:**
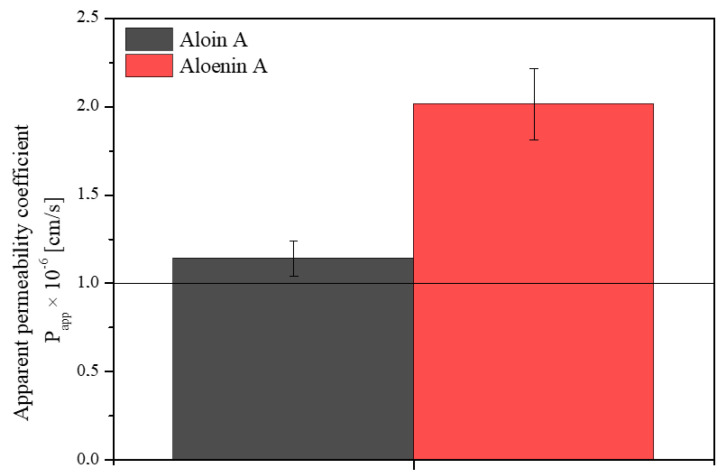
Apparent permeability coefficients for aloin A (black) and aloenin A (red) from lyophilized aloe gel using the SKIN model.

Knowing the health-promoting properties of the aloe gel water extract, it is worth considering whether its action is limited only to the surface effect or whether the active compounds can penetrate the skin barrier. The PAMPA-SKIN model was used to predict the transdermal penetration of compounds (aloin A and aloenin A). Figure 2 shows the apparent permeability coefficients for active ingredients. Both in the case of aloin A and aloenin A, P_app_ > 1 cm/s was obtained, which proves a high passive permeability through the skin barrier. However, aloin has been shown to be photocytotoxic when absorbed at a dose of 500 µM and irradiated with UV light, so a topical application must be limited to the skin surface or at a dose of 500 µM and irradiated with UV light and/or exposure to UV light should be avoided [39,40]. It is worth bearing in mind that the design of a pharmaceutical form should limit aloin’s absorption and allow it to show its action on the skin surface. Otherwise, due to a matter of safeness, an additional purification step should be added to the methodology to remove the aloin.

Many properties of the aqueous extract of aloe gel have been demonstrated, such as antioxidant, anti-hyaluronidase, and anti-tyrosinase effects, as well as an effect on cholinesterases. All these properties lead to the possibility of using the extract in the treatment of hard-to-heal diabetic wounds. It is essential to formulate an appropriate pharmaceutical form that, on the one hand, ensures the stability of the product and, on the other hand, limits the absorption of aloin, as described above. Therefore, the second part of the work aimed to create a semi-solid pharmaceutical form in the hydrogel form with the targeted release of active compounds. Hydrogels have been chosen as the pharmaceutical dosage form with the controlled release of active ingredients. Because of their ability to cleanse the wound by absorbing exudate as well as impurities, maintain the wound surface wet, and regulate the entry of water vapor and oxygen, hydrogels are regarded as good topical solutions for the treatment of chronic wounds [41]. Sodium alginate (formulation 1) and hydroxypropyl methylcellulose (HPMC) (formulation 2) were proposed as the base for hydrogels (Table 1). Alginate hydrogels offer exceptional features, such as a high water content, nontoxicity, soft consistency, biocompatibility, and biodegradability, making them ideal candidates for drug delivery [42]. Alginates are already described as an aloe vera gel carrier, where the possibility of diffusing the aloe vera gel by dissolving the film into the wound bed during swelling or when stimulating the healing process is indicated [43]. Meanwhile, HPMC was selected as a widely used cellulose derivative in controlled release products due to its thickening, gelling, and swelling properties [44], and it has also already been described as an aloe gel vehicle [45,46].

**Figure 3:**
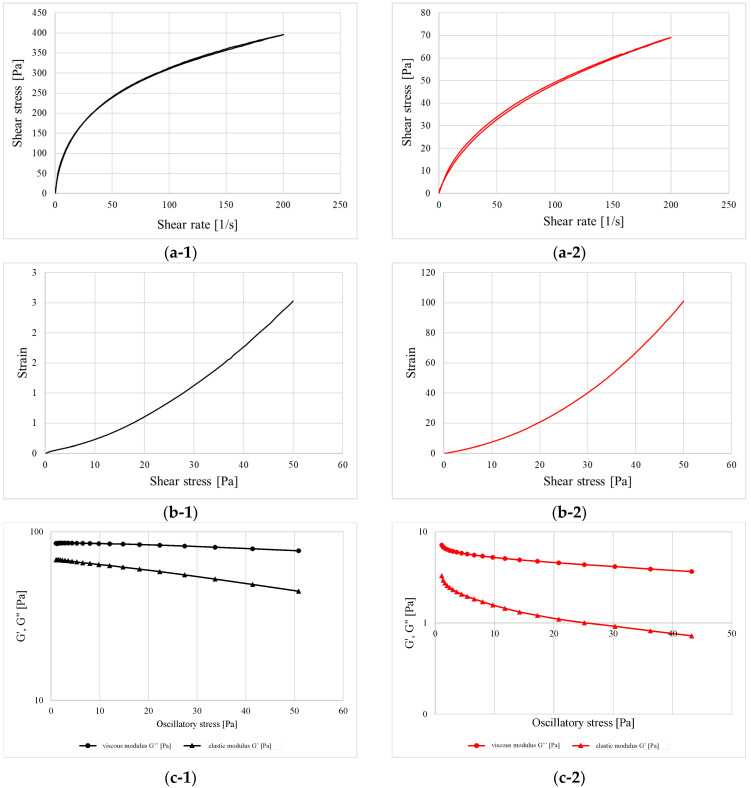
Flow curves obtained in controlled rate (CR) mode (**a-1**,**a-2**), flow curves obtained in controlled stress (CS) mode (**b-1**,**b-2**) and the oscillatory stress sweeping (**c-1**,**c-2**) for formulation 1 (black) and 2 (red).

Fresh-prepared alginate- and HPMC-based hydrogels have undergone rheological tests, divided into two parts: steady shear and oscillatory shear measurements (Figure 3). As can be observed in Figure 3(a-1,a-2), the shape of the flow curves indicated that both formulations revealed the shear-thinning properties with the loss of viscosity upon increasing the shear rate, which is typical for most polymer-based hydrogels and viscous liquids. From the values of shear stress reached at the maximum shearing rate, it can be concluded that the addition of sodium alginate contributed to the stiffening of the structure by more than five-fold when compared to the HPMC. The curves showed the best fitting to Ostwald−de Waele model, depicted by the equation: *τ* = *K* · *n*, where *n* is a power-law index [47]. Its value corresponds to the fluidity degree of a given material and represents the departure from Newtonian behavior (*n* = 1). When *n* reaches values beyond unity, the material is starts thinning upon the applied shearing force and when it exceeds the value of unity, the material starts shear thickening and reacts to the viscosity increase. In the first case, the lower the *n* value, the higher the deviation from Newtonian flow [48]. The K parameter, in turn, is described as the consistency coefficient and is equal to the shear stress at a shear rate of 1.0 s^−1^. As presented in Table 5, the consistency of the alginate-based sample turned out to be more than ten times greater than the HPMC-containing sample. This is the effect of both a higher polymer concentration but also the interaction of functional groups with the ions present in the formulation and the formation of cross-links. In this aspect, the HPMC is not capable of creating this type of connection, and the way of reacting to the applied force depends to the greatest extent on the concentration and the degree of chain entanglement [49].

The overlapping of the up- and down ramps on the plots a-1 and a-2, as well as data from Table 5, clearly shows that the thixotrophy of both gels was negligible and the structure, after being disrupted, rebuilds almost instantly. According to the CS mode measurements (Figure 3(b-1,b-2)), it can be stated that the samples possessed no apparent yield point, which can be concluded from the fact that even when applying the minimum amount of force, the formulations already began to deform and flow without putting up any significant resistance [50].

Figure 3(c-1,c-2) represent the behavior of samples upon increasing oscillatory stress. In both cases, the value of the viscosity modulus being higher than the elasticity modulus indicate that the samples do not contain a distinct three-dimensional polymer network and the structure is the result of a mutual entanglement of individual chains and other components. Therefore, the tested systems exhibit the characteristics of viscous liquids without a rigid internal structure.

**Figure 4:**
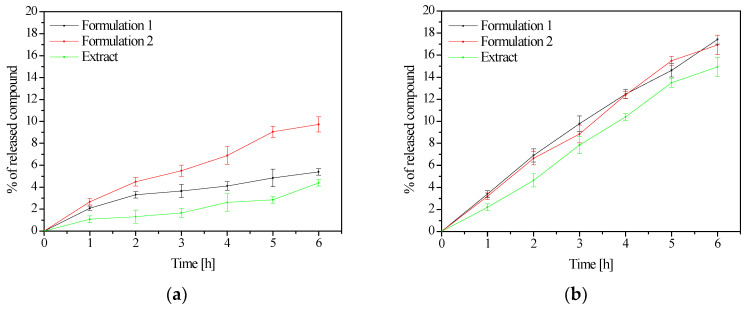
Released profiles of aloin A (**a**) and aloenin A (**b**) from formulation 1 and 2.

When analyzing the dissolution profiles of aloin, it was noted that its behavior changed depending on the formulation, while aloenin was released at a comparable degree from both forms (Figure 4). The release of analyzed standards did not reach 10% in the case of aloin A and 20% in aloenin A, which is a very low score. This is a significant discovery considering the need to retain aloin on the skin surface as described above. The slower drug release in the buffer phase for alginate matrices could be due to the formation of a more viscous and erosion-resistant ionic gel barrier [51]. Similarly, the slowed-down release of aloin from the HPMC matrix can be explained by the formation of an osmotic gradient which results in a quicker swelling rate of the polymer and a considerable increase in the thickness of the gel, consequently increasing the viscosity of the gel matrix and lowering the effective coefficient of drug diffusion [52]. There are no data in the literature showing release from a single alginate matrix or HPMC. However, Bialik-Wąs et al. proposed sodium alginate/poly(vinyl alcohol) (SA/PVA) hydrogel films, obtaining a prolonged release of Aloe vera up to five days. However, the method based on the polysaccharides was used, which did not contain the release behavior of individual active compounds [53]. The literature also describes the use of other polymers such as water-soluble and non-toxic polyvinyl alcohol (PVA) and carboxymethyl cellulose (CMC) for the formation of aloin hydrogels where 80% of aloin was released after 6 h [54]. On the contrary, aloin was not released at all from 1% gels based on Carbopol 974P and Sepigel 305, most likely by hydrogen bonds between the molecule and the aqueous environment [55]. The results of the research and literature data clearly indicate the significant impact of the selection of the hydrogel vehicle.

It is worth adding that, despite the low level of aloenin A release, the prepared formulations 1 and 2 showed comparable activity to the starting lyophilized aqueous gel. In the case of antioxidant activity (DPPH assay), IC50 was 34.24 ± 0.71 and 33.45 ± 1.49 mg/mL for formulation 1 and 2, respectively. Inhibition of hyaluronidase was 5.80 ± 1,12% and 6.02 ± 0.59%, whereas the inhibition of tyrosinase was 11.23 ± 2.32% and 12.56 ± 1.54%, for formulation 1 and 2, respectively. The obtained results do not differ statistically from those obtained for the starting lyophilized aqueous gel, which confirms the lack of influence of the hydrogel base on the activity of the finished formulation.

**Figure 5:**
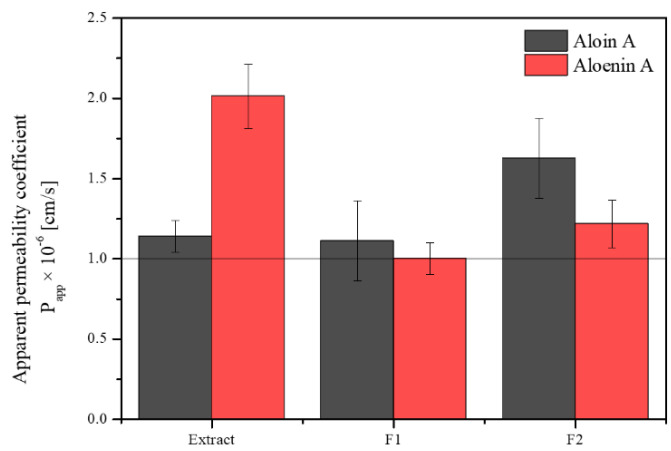
Apparent permeability coefficients for aloin A (black) and aloenin A (red) from formulation 1 (F1) and formulation 2 (F2) using the SKIN model.

Finally, the permeation of active compounds from the hydrogels was also assessed (Figure 5). A decrease in the penetration of both active compounds was observed, more significantly in the case of aloenin. Again, this may be due to sticky gel formation and limited diffusion from the form. It is worth noting that both hydrogels limit the release and penetration of aloin. In the case of aloin, it is desirable to minimize its absorption to restrict the drug to the diseased area of application, avoiding the potential side effects associated with absorption. Aloin skin permeation was restricted (higher for formulation 1) because of its high affinity for the vehicle, which causes not only a slow release but also poor skin transfer [55]. It would also be appropriate to add an additional purification step to the extract to completely eliminate the aloin.

The last stage included stability studies of the developed formulations. The changes in the concentrations of both active compounds were assessed on days 0, 10, and 20 for both the lyophilized extracts and formulations 1 and 2 (Table 6). In no case was the compound content maintained at 100 ± 10% at 30 °C. Therefore, refrigerated storage is worth considering, as also indicated by Suriati et al. [56]. Importantly, no changes in color or consistency were observed during storage. Moreover, the addition of HPMC has little effect on the difference in the formulation stability, while the addition of sodium alginate significantly reduces this stability.

Taking into account all the studies carried out, the HPMC-based hydrogel (formulation 2) can be indicated as the one with a targeted release of active substances and greater stability. Therefore, it is worth developing this pharmaceutical form further.

## 4. Conclusions

Both the aqueous gel from *Aloe arborescence* as well as the hydrogel as a pharmaceutical dosage form containing aqueous gel *Aloe arborescence* did not involve the use of organic solvents. For the aqueous gel from *Aloe arborescence*, significant biological activity was confirmed, indicating the obtained leaf processing as a valuable starting substrate for the hydrogel formulation. The prepared hydrogel meets the functional criteria in terms of the maintenance required activity and rheological parameters, therefore, it can be suggested that the hydrogel with *Aloe Arborescence* is a promising approach form of the treatment of skin wounds.

## Figures and Tables

**Table 1:** Chemical composition of hydrogels.

	Formulation 1 (F1)	Formulation 2 (F2)
Lyophilized aloe gel	1.50 g (3%)	1.50 g (3%)
Hyaluronic acid	0.75 g (1.5%)	0.75 g (1.5%)
Sodium alginate	1.50 g (3%)	-
Hydroxypropyl methylcellulose (HPMC)	-	1.00 g (2%)
Water	up to 50.00 g	up to 50.00 g

**Table 2:** The antioxidant effect of water extracts of lyophilized aloe gel.

	DPPH IC_50_ [mg/mL]	CUPRAC IC_0.5_ [mg/mL]
Water extract of lyophilized aloe gel	35.50 ± 0.57	22.35 ± 0.35

**Table 3:** Inhibition of hyaluronidase and tyrosinase activity.

	% Inhibition of Hyaluronidase	% Inhibition of Tyrosinase
Water extract of lyophilized aloe gel at concentration 66.67 mg/mL	7.03 ± 0.43	14.43 ± 1.03

**Table 4:** Inhibition of AChE and BChE activity.

	AChE Inhibition IC_50_ [mg/mL]	BChE Inhibition IC_50_ [mg/mL]
Water extract of lyophilized aloe gel	265.37 ± 1.87	120.87 ± 2.37
Galantamine	0.02 ± 0.01	0.13 ± 0.01

**Table 5:** The calculated rheological parameters from the course of the flow-curves.

	Ostwald−de Waele			Thixotropy
	Power Law Index (*n*)	Consistency (*K*) [Pa∙s^n^]	Correlation Coefficient	
Formulation 1	0.384 ± 0.005	51.92 ± 1.97	0.9979	165.75 ± 1.20
Formulation 2	0.533 ± 0.006	4.20 ± 0.00	0.9989	180.80 ± 5.94

**Table 6:** Stability of aloin A and aloenin A.

Formulation	Days	Aloin A	Aloenin A
		mg of Aloin A per g Dry Weight	mg of Aloenin A per g Dry Weight
Lyophilized extract	0	1.49 mg (100.00%)	1.19 mg (100.00%)
	10	1.22 ± 0.05 mg (81.88 ± 3.36%)	0.74 ± 0.02 mg (62.18 ± 1.68%)
	20	0.75 ± 0.03 mg (50.34 ± 2.01%)	0.75 ± 0.03 mg (63.03 ± 2.52%)
Formulation 1	0	0.14 mg (100.00%)	0.16 mg (100.00%)
	10	0.11 ± 0.01 mg (78.57 ± 7.14%)	0.06 ± 0.01 mg (37.50 ± 6.25%)
	20	0.05 ± 0.01 mg (35.71 ± 7.14%)	0.04 ± 0.01 mg (25.00 ± 6.25%)
Formulation 2	0	1.40 mg (100.00%)	1.02 mg (100.00%)
	10	1.07 ± 0.04 mg (76.43 ± 2.86%)	0.90 ± 0.02 mg (88.24 ± 1.96%)
	20	1.01 ± 0.03 mg (72.14 ± 2.14%)	0.81 ± 0.03 mg (79.41 ± 2.94%)

## Data Availability

Not applicable.
